# P-2280. Community-Acquired Pneumonia in Immunosuppressed Patients Admitted to the ICU

**DOI:** 10.1093/ofid/ofae631.2433

**Published:** 2025-01-29

**Authors:** André Emilio Viñán Garcés, Natalia Sanabria-Herrera, Sara Duque, Esteban Garcia-Gallo, Gabriela Guerrón-Gómez, Luis Felipe F Reyes

**Affiliations:** Unisabana Center for Translational Science, School of Medicine, Universidad de La Sabana, Chía, Colombia, Chía, Cundinamarca, Colombia; Clínica Universidad de La Sabana, Chía, Colombia, Bogota, Distrito Capital de Bogota, Colombia; Pandemic Sciences Institute, University of Oxford, Oxford, UK, Oxford, England, United Kingdom; Pandemic Sciences Institute, University of Oxford, Oxford, UK, Oxford, England, United Kingdom; Clínica Universidad de La Sabana, Chía, Colombia, Bogota, Distrito Capital de Bogota, Colombia; Universidad de La Sabana, Chía, Cundinamarca, Colombia

## Abstract

**Background:**

Severe community-acquired pneumonia (sCAP) remains a crucial infectious cause of global mortality, accounting for a significant proportion of ICU admissions. Immunosuppressed patients frequently develop sCAP; however, the clinical characteristics and clinical outcomes of patients admitted to the ICU due to sCAP are not clear. Here, we attempted to bridge this gap in the literature.

Figure 1

**Figure:**
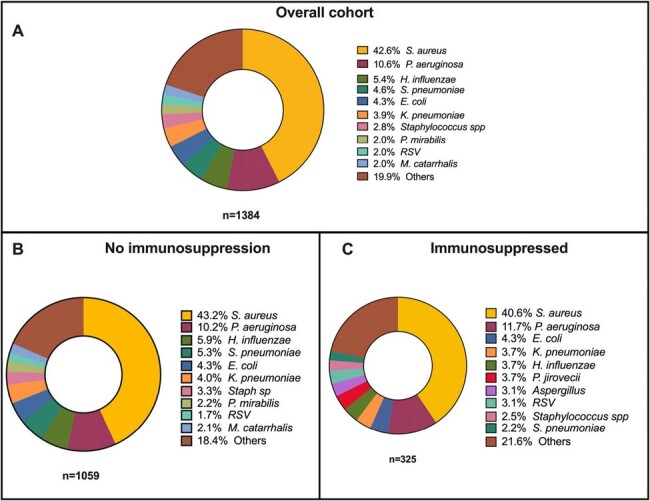

Isolated microorganisms in respiratory samples. A. General cohort; B. No immunosuppression risk factors group; C. Immunosuppression risk factors group

**Methods:**

This secondary analysis of the prospective MIMIC-IV database included adults admitted to the ICU with CAP diagnosis. Patients were stratified based on the presence or absence of immunosuppression. Bivariate, multivariate, and survival analyses were conducted to identify factors associated with mortality within the immunosuppressed and their impact on hospital mortality.

Figure 2

**Figure:**
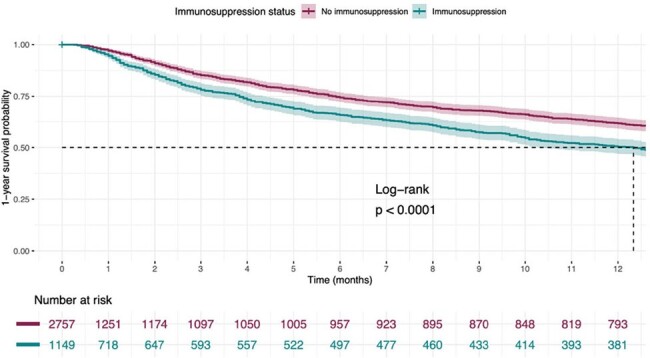

Kaplan-Meier survival curve of 1-year after discharge survival probability stratified by immunosuppression status. Patients with and without immunosuppression are represented, the first group with 1 (blue) and the last with 0 (red).

**Results:**

A total of 4742 patients were included, with 1502/4742 (32%) being immunocompromised. The mean age (SD) was 66 ±16.1years, with 2627/4742 (55.4%) males. A total of 1384 respiratory isolations were obtained with Staphylococcus aureus (43.2% vs. 40.6%; p= 0.45) and Pseudomonas aeruginosa (10.20 % vs 11.69 %; p= 0.39) as the most representative in both groups (Figure 1). Immunosuppressed patients presented both higher Charlson comorbidity index (7.12, SD 3.14 vs. 4.97, SD 2.59; p < 0.01) and infection severity by the SAPS II score (39, [31-49] vs 37, [ 29-46]; p < 0.01). Immunosuppression was associated with increased mortality rates (IH: OR 1.99 [95% CI: 1.63-2.43]; 6 M: OR 1.79 [95% CI 1.52-2.12]; 12 M: OR 1.93 [95% CI 1.64-2.28]; p < 0.01) and both solid malignancy and infection severity were positively correlated with mortality (Table 1). Immunosuppressed patients had a lower survival rate even after adjusting for disease severity (Figure 2).

Table 1

**Figure:**
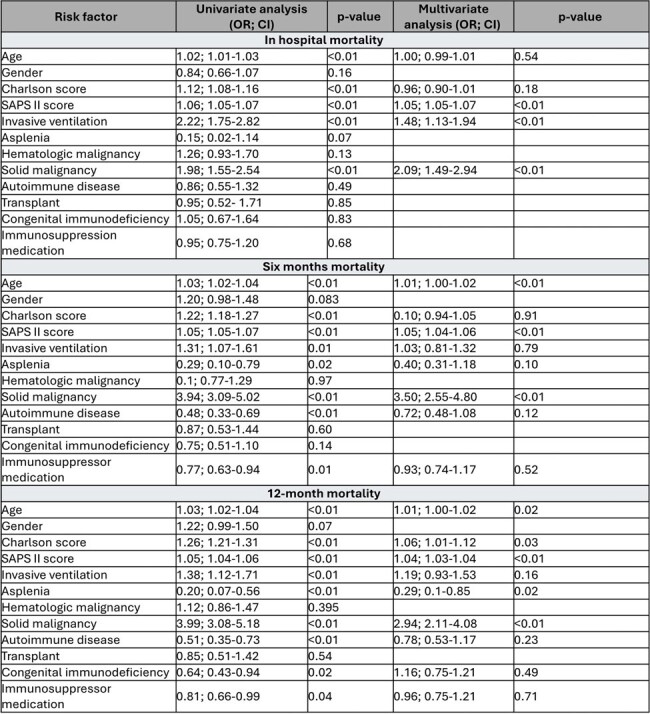

Univariate and multivariate analysis of risk factors associated with in-hospital, 6-month, and 12-month deaths in the immunosuppressed population.

**Conclusion:**

Immunosuppression was independently associated with mortality and lower survival in sCAP patients. An individualized patient risk approach is necessary as immunosuppression affects mortality differently than immunocompetent patients.

**Disclosures:**

All Authors: No reported disclosures

